# Epidemiological changes in meningococcal meningitis in Niger from 2008 to 2011 and the impact of vaccination

**DOI:** 10.1186/1471-2334-13-576

**Published:** 2013-12-06

**Authors:** Jean-Marc Collard, Bassira Issaka, Maman Zaneidou, Stéphane Hugonnet, Pierre Nicolas, Muhamed-Kheir Taha, Brian Greenwood, Jean-François Jusot

**Affiliations:** 1 Centre de Recherche Médicale et Sanitaire, Niamey, Niger; 2 Direction de la Surveillance et de la Riposte aux Epidémies, Ministère de la Santé Publique, Niamey, Niger; 3 World Health Organization, Geneva, Switzerland; 4 WHO Collaborative Center for the meningococcus in IMTSSA (Institut de Médecine Tropicale du Service de Santé des Armées), Marseille, France; 5 Institut Pasteur, WHO Collaborating Centre for Reference and Research on Meningococci, Paris, France; 6 Faculty of Infectious and Tropical Diseases, London School of Hygiene and Tropical Medicine, London, UK; 7 634 Boulevard de la Nation, YN034, PO Box 10887 Niamey, Niger; 8 Current address: Scientific Institute of Public Health, Brussels, Belgium

**Keywords:** *Neisseria meningitidis* serogroup A and W, Meningitis, Niger, Vaccination, MenAfriVac®, Sequence-type lineage evolution, Immunization

## Abstract

**Background:**

The epidemiology of bacterial meningitis in the African ‘meningitis belt’ changes periodically. In order to design an effective vaccination strategy, we have examined the epidemiological and microbiological patterns of bacterial meningitis, and especially that of meningococcal meningitis, in Niger during the period 2008–2011. During this period a mass vaccination campaign with the newly developed meningococcal A conjugate vaccine (MenAfriVac®) was undertaken.

**Method:**

Cerebrospinal fluid samples were collected from health facilities throughout Niger and analysed by culture, seroagglutination and/or speciation polymerase chain reaction, followed by genogrouping PCR for *Neisseria meningitidis* infections. A sample of strains were analysed by multi-locus sequence typing.

**Results:**

*N. meningitidis* serogroup A cases were prevalent in 2008 and 2009 [98.6% and 97.5% of all *N. meningitidis* cases respectively]. The prevalence of serogroup A declined in 2010 [26.4%], with the emergence of serogroup W Sequence Type (ST) 11 [72.2% of cases], and the serogroup A meningococcus finally disappeared in 2011. The geographical distribution of cases *N. meningitidis* serogroups A and W within Niger is described.

**Conclusion:**

The substantial decline of serogroup A cases that has been observed from 2010 onwards in Niger seems to be due to several factors including a major polysaccharide A/C vaccination campaign in 2009, the introduction of MenAfriVac® in 10 districts at risk in December 2010, the natural dynamics of meningococcal infection and the persistence of serogroup A sequence-type 7 for about 10 years. The emergence of serogroup W strains suggests that there may be a need for serogroup W containing vaccines in Niger in the coming years.

## Background

*Neisseria meningitidis* (Nm) is responsible for frequent outbreaks of meningococcal meningitis in the African ‘meningitis belt’, a sub-Saharan zone which stretches from Ethiopia to Senegal. Epidemics show marked seasonality, peaking during the dry, hot season [1] and they can cause many deaths (mortality around 10%) and residual disability. During major epidemics, attack rates can range from 100 to 800 per 100 000 population.

Nm serogroup A (Nm A) is the main cause of epidemics in Africa. However, serogroup C was responsible for epidemics in Nigeria in the 1970s [2] and in Upper Volta (now Burkina Faso) in the 1980s [3], serogroup W sequence type (ST)-11 caused severe epidemics in Burkina Faso in 2002 and 2003 [4,5], and serogroup X caused an outbreak in Niger in 2006 [6]. A strategy of reactive immunization with an anti-meningococcal A/C polysaccharide vaccine has been used for many years to control epidemics in the African meningitis belt, based on WHO recommendations [7]. This approach has mitigated the extent of meningitis outbreaks but it has not prevented the continuing occurrence of large outbreaks of the disease. The reactive vaccination strategy relies on early detection of outbreaks followed by mass vaccination with a polysaccharide vaccine adapted to the circulating serogroup, once an incidence threshold has been crossed. Polysaccharide vaccines provide only short-term protection in children and have little or no impact on pharyngeal carriage [8]. A newly developed meningococcal A conjugate vaccine, MenAfriVac®, was introduced in 2010 in Burkina Faso, Mali and Niger through mass vaccination campaigns directed at those aged 1-29 years. Initial findings from Burkina Faso and Chad suggest that this vaccine provides some herd protection, by preventing pharyngeal carriage, as well as protecting directly vaccinated individuals [9,10], as seen following the introduction of a monovalent meningococcal C conjugate vaccine in the United Kingdom [11]. The aim of the MenAfriVac® programme is to eliminate large meningitis outbreaks in the 26 most severely affected countries of sub-Saharan Africa.

The purpose of the study reported here was to study the circulation of Nm A and Nm W in Niger since 2008, and to examine the current epidemiological situation in relation to the availability of polysaccharide vaccines against Nm W. We have also tried to correlate the marked decrease in the number of cases caused by Nm A, and the increased number due to Nm W, with data on vaccination and to speculate on the evolution of sequence-type lineages and naturally acquired immunity in the study population.

## Methods

### Ethics statement

Cerebrospinal fluid (CSF) samples were collected from patients with suspected meningitis by clinicians, with the patient’s or their family’s oral consent, according to the national guidelines for routine clinical care in Niger. Aliquots of CSF remaining after routine CSF examination by health facilities for case management were sent on a voluntary basis to the Centre de Recherche Médicale et Sanitaire (CERMES) to meet the WHO guidelines of enhanced microbiological surveillance of meningitis. Confidentiality on patients’ identity was guaranteed.

### Introduction of MenAfriVac®

MenAfriVac®, a meningococcal A (MenA) tetanus toxoid conjugate vaccine, developed by the Meningitis Vaccine Project (MVP) (http://www.meningvax.org/), was introduced on a pilot scale in September 2010 in Niger (1 district). The first district to be vaccinated was Filingué, targeting 392,211 individuals aged 1-29 years (data from the Direction of Surveillance and Response to Epidemics (DSRE), Ministry of Public Health (MoPH), Niger). In December 2010, MenAfriVac® was introduced on a larger scale in Niger. Eight districts of the Tillabery and Niamey Regions, and 2 districts from the Dosso Region were vaccinated covering 3.1 millions of individuals. In 2011, Niger completed the last phase of their own vaccination campaigns (7.4 million individuals), boosting the total number vaccinated to almost 10.9 million.

### Epidemiological surveillance

Epidemiological surveillance in Niger is undertaken by the Direction of Surveillance and Response to Epidemics (DSRE), MoPH. Quantitative data on incidence and the mortality per age class for all clinically suspected cases of meningitis who meet a standard case definition are notified weekly to the MoPH. This clinical surveillance is coupled to microbiological surveillance performed by CERMES using CSF samples collected throughout Niger.

### Collection of CSF

In Niamey, between November and June (the meningitis season), aliquots of CSF kept at room temperature after routine CSF examination by national and regional hospitals for case management were collected by CERMES. Additionally, during the same period from 2003-2010, frozen CSF samples were collected monthly by CERMES from hospitals within a radius of 300 km from Niamey (Dosso and Tillabéry Regions). This collection was stopped at the time of the implementation of enhanced case by case surveillance in 2011. Refrigerated or frozen CSF samples, and occasionally CSF-inoculated trans-isolates (TI), were sent on a voluntary basis to CERMES or DSSRE by mandated transport companies from the rest of the country.

### Bacteriological and molecular analysis of CSF

Laboratory confirmation of meningococcal meningitis was performed at CERMES using culture and/or PCR techniques on CSF or TI as described previously [12]. CERMES provided information weekly to the DSRE on the number of confirmed cases, their aetiology, sampling date and geographical distribution to aid decisions on epidemic control.

### Multi-locus sequence typing

A representative sample of strains of *N. meningitidis* was sent every year from 2002 to the WHO collaborative centre (WHOCC) for the meningococcus in IMTSSA (Marseille, France) for multi-locus sequence typing (MLST). In 2011, strains were sent to the new WHOCC for the meningococcus at the Pasteur Institute, Paris, France. DNA-PCR-amplified fragments from seven housekeeping genes (*abcZ, adk, aroE, fumC, gdh, pdhC* and *pgm*) were sequenced and their allelic profiles compared to the existing alleles available on the Internet Multilocus sequence typing data bank (http://www.mlst.net) to determine their sequence type [13].

### Data management and analysis

Descriptive statistics were used to describe the epidemiology of meningococcal meningitis using both data from epidemiological surveillance provided by the MoPH and from microbiological surveillance performed by CERMES.

## Results

### Surveillance data

**Figure 1:**
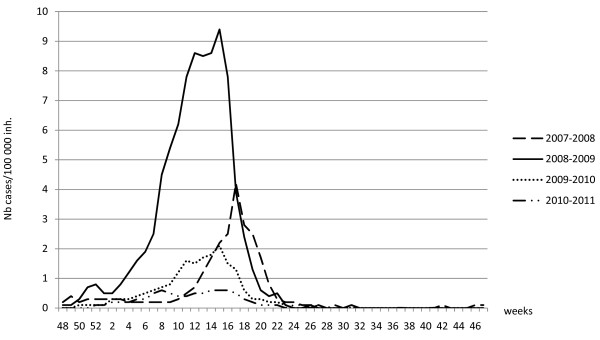
Attack rates of meningitis in Niger during the years 2008 to 2011 (data collected by the Direction of Surveillance and Response to Epidemics – Ministry of Public Health).

**Figure 2:**
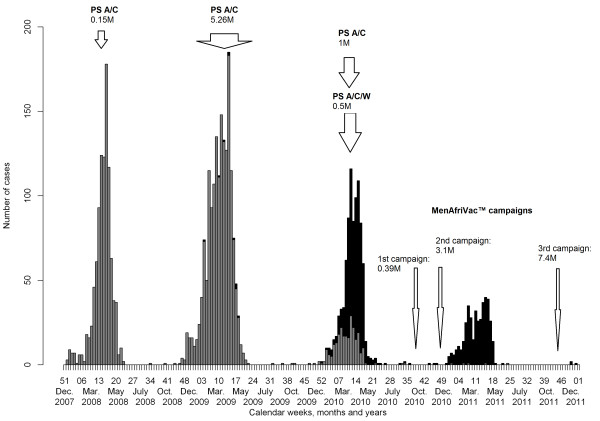
**Epidemic curves of confirmed *N. meningitidis *serogroups A (grey histograms) and W (black) cases on a weekly scale detected from 2008 to 2011.** Vaccinations with polysaccharide (PS) bivalent A/C and trivalent A/C/W vaccines are indicated (with the number of administered doses). The three successive MenAfriVac® campaigns are also shown.

From 1^st^ January 2008 to 31^st^ December 2011, the MoPH of the Republic of Niger reported a total of 22, 046 suspected cases of meningitis with a case fatality rate (CFR) of 5.7% (Table 1, Figure 1). A major epidemic in 2009 accounted for 63.2% of the total cases but this outbreak had the lowest CFR (4.2%). Microbiologic analyses of all CSF samples obtained during these four years are presented in Table 1 and shown on a weekly scale in Figure 2. In the 2008 and 2009 meningitis seasons, a large majority of meningococcal infections were due to Nm A (98.6%; N = 1067 and 97.5% ; N = 1654 respectively). Nm W emerged in 2010 (72.2% of isolates; N = 665) while the percentage of Nm A isolates declined (26.4%; N = 243), and in 2011 there was a strong predominance of Nm W isolates (98.1%; N = 402) with only 5 isolates of Nm A. The increase in Nm W meningitis cases was accompanied by an increase in the CFR (from 6.7% and 4.2% respectively in 2008 and 2009 to 12.2% in 2011). Two of the five cases of Nm A meningitis detected in 2011 in zones vaccinated with MenAfriVac® concerned young males aged 12 and 25 years that had not been vaccinated. The other three cases came from districts where MenAfriVac® had not been distributed at that time that they became ill (Keita and Matameye in the central part of the country and Agadez in the north). Nm X cases were infrequent with only 22 PCR-positive CSF samples obtained over the four-year period, 15 of which were detected in 2009. Numbers of cases of *Streptococcus pneumoniae* meningitis remained relatively constant over the observation period with 117, 78, 93 and 70 cases respectively detected in 2008, 2009, 2010 and 2011 respectively. Pneumococcal conjugate vaccination will be introduced in Niger likely in 2015. Fifty-four percent (N = 5393) of CSF samples remained without a bacteriological aetiology after culture and PCR.

**Table 1:** Results from the epidemiological and microbiological surveillance of meningitis in Niger (2008-2011)

**Year**	**Notified cases**	**Deaths**	**CFR**	**CSF collected**	**% CSF collected/declared cases**	**Nm (all types)**	**Nm A**	**Nm W**	**Nm X**	**Other serog.**	**Non-typ. Nm**	**Spn**	**Hi**	**Other**	**Negative CSF**
**2008**	3851	256	6.65	2716	70.5	1082	1067 (98.6%)	0	5	0	10	117	33	23	1461
**2009**	13943	589	4.22	3915	28.1	1696	1654 (97.5%)	11	15	1 (Y)	15	78	12	66	2063
**2010**	2908	251	8.63	2384	82.0	921	243 (26.4%)	665 (72.2%)	1	0	12	93	22	26	1322
**2011**	1344	164	12.20	1041	77.5	410	5	402 (98.1%)	1	0	2	70	2	12	547
**Total**	**22046**	**1260**	**5.71**	**10056**	**45.6**	**4109**	**2969**	**1078**	**22**	**1**	**39**	**358**	**69**	**127**	**5393**

Epidemiological data are from the Direction of Surveillance and Response to Epidemics (DSRE), Ministry of Public Health; Microbiological data from CERMES.

CSF, cerebrospinal fluid; Nm, *Neisseria meningitidis*, CFR, case fatality rate.

Spn, *Streptococcus pneumoniae*; Hi, *Haemophilus influenzae*.

‘Other’ are other aetiologies (i.e. *Salmonella* sp.), contaminated or broken samples.

**Figure 3:**
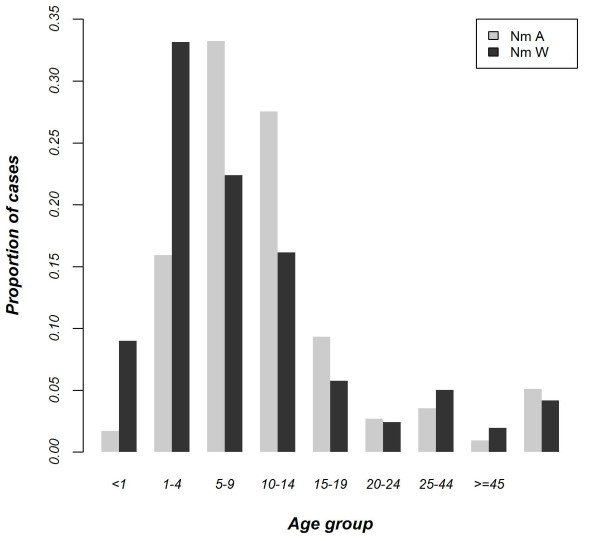
**Age groups affected by *N. meningitidis *** **serogroups A (N = 2964) and W (N = 1077) from 2008 to 2011.**

The mean ages of confirmed cases of serogroup A meningitis (N = 2969), and serogroup W meningitis (N = 1078), were 10.3 (SD = 7.8), and 8.9 (SD = 10.3) years respectively (t-test = 3.95, df = 1490, p < 0.0001). The age groups most affected were 5-9 years and 1-4 years for serogroups A and W respectively (Figure 3). The numbers of serogroups A and W confirmed cases in those less than 2 years were 84 (2.97%) and 168 (16.28%), respectively.

### MLST Molecular typing

Most of the Nm A strains analysed (N = 141) by MLST during the 2002-2011 period belonged to ST-7 (N = 131; 92.9%) (Table 2), except for nine ST-2859 isolates (6 in 2007, 2 in 2008 and 1 in 2009) and one ST-5788 isolate (obtained in 2006). The strains belonging to ST-2859 all originated from Niamey, whereas the other ST-7 strains were isolated from all parts of the country. Nm W strains isolated in 2010 and 2011 (N = 30) and analysed by MLST belonged to ST-11. Nm W ST-11 strains were also isolated in 2002 (N = 4) and 2003 (N = 5). From 2004 to 2009 all Nm W strains analysed were ST-2881 (N = 22).

**Table 2:** Evolution of meningococcal Sequence-type (ST) and Clonal Complexes (cc) of serogroups A and W from meningitis cases in Niger from 2002 to 2011

***Serogroup***	***Year***	***ST***
A	2002	**ST-7** (N = 11) (cc5)
	2003	**ST-7** (N = 22) (cc5)
	2004	**ST-7** (N = 13) (cc5)
	2005	**ST-7** (N = 16) (cc5)
	2006	**ST-7** (N = 11) (cc5) and **ST-5788** (N = 1) (cc5)
	2007 (Nov)	**ST-7** (N = 21) (cc5) and **ST-2859** (N = 6) (cc5)
	2008	**ST-7** (N = 7) (cc5) and **ST-2859** (N = 2) (cc5)
	2009	**ST-7** (N = 27) (cc5) and **ST-2859** (N = 1) (cc5)
	2010	**ST-7** (N = 3) (cc5)
W	2002	**ST-11** (N = 4) (cc11)
	2003	**ST-2881** (N = 7) (cc175) and **ST-11** (N = 5) (cc11)
	2004	**ST-2881** (N = 4) (cc175)
	2005	**ST-2881** (N = 7) (cc175)
	2006	**ST-2881** (N = 2) (cc175)
	2007	-
	2008	**ST-2881** (N = 2) (cc175)
	2009	-
	2010	**ST-11** (N = 13) (cc11)
	2011	**ST-11** (N = 17) (cc11)

Data are from the WHO Collaborating Center on Meningococci at Institut de Médecine Tropicale du Service de Santé des Armées (IMTSSA), Marseille, France before 2011 and from the Pasteur Institute, Paris, France since 2011.

### Geographical analyses

**Figure 4:**
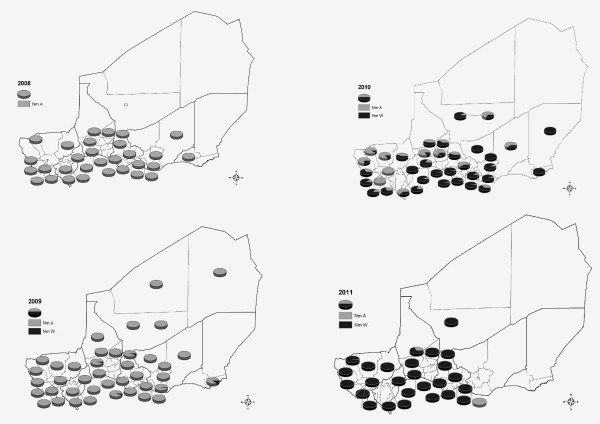
**Geographical extension of *N. meningitidis *serogroup A (Nm A) from 2008 to 2009, its decline from 2010 to 2011 and, in parallel, the extension of Nm W in 2010 and 2011.** The proportions of Nm A cases/Nm W cases per district are represented by coloured pie charts.

A map of the Regions (or Departments) and Districts (or Arrondissements) of Niger can be accessed at http://commons.wikimedia.org/wiki/Atlas_of_Niger. Figure 4 shows the geographic extension and decline of Nm A in Niger between 2008 and 2011, and the extension of Nm W since 2010.

**2008:** Nm A cases, representing 98.6% of the total Nm cases, were concentrated in the southern and central parts of the country (Dosso, Tahoua, Maradi and Zinder regions), along the border with Nigeria.

**2009:** Nm A cases dominated again (97.5%) with very few cases of other serogroups (15 Nm X, 11 Nm W, 1 Nm Y, 15 non groupable (NG)-Nm) being detected among 3915 CSF samples analyzed. Nm A cases were spread over almost the whole country. During the epidemic season twenty-five of the 42 health districts had an attack rate of suspected cases over 10/100,000/week (epidemic threshold) and 11 districts an attack rate over 5/100,000/week (alert threshold). During the epidemic peak, attacks rates reached values up to 33.4 and 27.6/100,000/week in Madarounfa and Magaria districts respectively.

**Figure 5:**
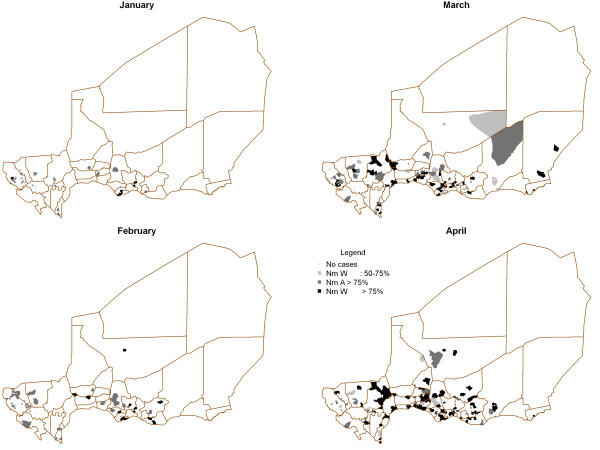
Spatio-temporal distribution of the percentage of Nm A and Nm W in health centre catchment areas (HCCA) of Niger between January and April 2010.

**2010:** Nm A cases (26.4% of all meningococci) were still distributed across almost all districts (32/42), but the number of confirmed cases had decreased remarkably (N = 243) whereas Nm W cases increased dramatically in number (N = 665 cases; 72.2%) and were recorded in 35/42 districts. Nm A cases were concentrated in the western part of the country whereas the highest numbers of Nm W cases were observed in the southern and central parts of the country (mainly in Maradi and Madarounfa districts). To investigate the geographical distribution of the mixed epidemics of 2010, the distribution of serogroups A and W by month by health centre catchment area (HCCA) was determined. Figure 5 showed that in January 2010, Nm W cases began to emerge in HCCA of three districts, two located at the borders with Nigeria and one located at the border with Burkina Faso. Nm A cases were detected in HCCA of 11 districts. In February, Nm A remained the predominant serogroup and cases were present in HCCA of 12 districts whereas Nm W spread to HCCA of 9 districts located in the centre of Niger. In March, Nm W continued to be detected to the Western and Eastern region of Niger while Nm A remained predominant in HCCA of 10 districts located in the Western part of the country. April 2010 showed a clear predominance of Nm W in HCCA of 26 districts.

**2011:** Nm W cases (97.8%) were recorded predominantly in the western and central parts of the country (except for the district of Matameye). About 50% of the Nm W cases originated from the region of Tillabery vaccinated with MenAfriVac® in December 2010. No cases were reported from the eastern part of the country (Magaria, Zinder, Tanout, Mirriah, Maine Soroa, Nguigmi and Diffa districts) bordering Nigeria and Chad.

### Vaccination

In 2008, in response to the epidemic, 146,854 doses of polysaccharide (PS) A/C vaccine were administered in the districts of Filingue and Say, with coverage of 52.4% and 54.7% of the target population respectively.

In 2009, in response to the epidemic, but also to prevent outbreaks in some districts considered at risk, PS A/C vaccines were used to immunize a total of 5,904,978 subjects across the whole country with a vaccination coverage (mean per district) of 52.4% ±39% for the target population (including non-vaccinated districts). Coverage in vaccinated districts varied between 14% and 134% (population estimated on the last demographic census performed in 2001 explains values > 100%). The two regions the most at risk, Maradi and Zinder, had vaccination coverages of 64.2% and 80.4% respectively. However, other regions, such as Tahoua, were barely vaccinated (13.8%) with great variations between districts.

In 2010, in response to the epidemic due to Nm W, 509,621 doses of the trivalent PS A/C/W vaccine were distributed in the districts of Agadez, Maradi, Madarounfa, Madaoua and Zinder with vaccination coverage varying between 50% and 110%. An additional 1,062,577 doses of the PS A/C vaccine were administered, including 540,640 doses for the Tahoua region, increasing the general vaccination Nm A coverage of the region to 38%. In addition to vaccination responses, preventive vaccination campaigns with the MenAfriVac® vaccine were organized in 2010 and 2011 as explained in the Methods section (Figure 1).

## Discussion

Nm A has been the most prevalent cause of meningococcal meningitis in Niger since the start of microbiological surveillance in 1981, except in 1997 and 2006 when serogroup X [6,14] predominated. Serogroup X cases have not reemerged in Niger although observed in large numbers in Burkina Faso in 2011 [15].

The incidence of Nm A declined in Niger during the year 2010, the strain being replaced gradually by Nm W, and meningococci of this serogroup almost disappeared in 2011 (only five cases). This decline took place despite the fact that only 11 of 42 districts had received the conjugated vaccine (corresponding to 9.9 million of a total of 15.2 million inhabitants or 3.1 million of the 6.3 million target population for vaccination) in 2010.

There are several potential reasons which could account for the disappearance of Nm A from the whole country. The primary reason is likely to be the fact that meningococcal epidemics have a natural dynamic in the meningitis belt, as already described by Lapeyssonnie in 1963 [16], and there was a general decline in serogroup A colonization and disease in the meningitis belt prior to the introduction of the serogroup A conjugate vaccine in 2010 [15]. The persistence of Nm A Sequence-Type (ST)-7 for about 10 years in Niger (Table 2) may have contributed to the disappearance of the serogroup A meningococcus from the country around 2010 due to a build-up of natural immunity to this strain across the country during this period. Although the number of strains typed by MLST in our study was relatively low due to logistic constraints, and this could introduce a bias, we believe that our MLST results provide a reasonably representative picture of the different ST circulating during the epidemic seasons. Between 1995 and 1998, all Nm A isolates from Niger typed by MLST belonged to ST-5, from 1999 to 2001 to ST-7 (differing from ST-5 only at the *pgm* locus) [17], and from 2002 most of the isolates were ST-7 [18,19]. Serogoup A meningococci are largely clonal and all African isolates typed by the WHO Collaborating Centers for Reference and Research on Meningococci were remarkably homogeneous in their PorA (P1.20,9) and FetA (F3-1) sequences [20]. Hubert *et al.* also observed a remarkable antigenic stability of the PorA, PorB, and FetA proteins, but with occasional allelic exchange of *opa* genes, over years in Nm A isolates from Ghana and Burkina Faso [21]. According to Bart *et al.*[22], serogroup A meningococci do not persist in areas for more than a few years because they do not have the opportunity to accumulate genetic variants indefinitely and their survival mainly depends on geographic spread. Moreover, based on the time separating the ST-5 from the ST-7 outbreaks in Chad and Sudan, and the time taken for the spread of ST-5 from East Africa to Senegal and Guinea Bissau, Nicolas *et al.*[23] speculated that ST-7 might continue to spread until 2008 to 2009; our findings support this prediction.

Another important factor contributing to the disappearance of serogroup A cases of meningitis from Niger may have been the massive vaccination of the population with the PS A/C vaccine during the previous three years, especially during the large outbreak in 2009. There is good evidence that PS meningococcal vaccines have little impact on carriage in the African meningitis belt [24,25] and thus their widespread deployment is unlikely to prevent transmission or to reduce the reservoir of infection. However, high vaccine coverage rates will result in a marked reduction of cases among vaccinated people, even if some transmission of the epidemic strain continues within the population. This massive vaccination campaigns with the PS vaccine may have contributed to the drastic decrease of Nm A cases but this is unlikely to be the only reason. Vaccination with MenAfrivac®, which began in December 2010 in the 11 districts of the Western part of Niger most affected by Nm A that year, may have helped to reduce the transmission of Nm A, at least in this part of the country and as found in Burkina Faso and Chad [9,10].

Nm W has circulated in Africa for decades [26] but rarely caused outbreaks until the large epidemic in Burkina Faso in 2002 [27] caused by a sequence-type 11 serogroup W meningococcus, likely introduced by Hajj pilgrims returning in 2000 from Mecca and Madina (Saudi Arabia). In 2002-2003, molecular typing of the sporadic meningitis cases due to Nm W in Niamey showed two STs, ST-11 and ST-2881, the latter a new ST differing at six of the seven MLST typing loci (Table 2) [18]. Between 2004 and 2008, all Nm W strains typed (N = 15) belonged to the new ST-2881. Nm W strains of ST-11 have emerged again in Niger in 2010, causing a small epidemic (72.2% of all meningococci) [28]. As shown in Figure 5, the onset of epidemics due to Nm W strains started in the HCCA of three districts, two located at the borders with Nigeria and one located at the border with Burkina Faso, suggesting that Nm W strains were already present in the population and widespread in Niger, making diffusion from a single point unlikely [29]. In 2011, all Nm W strains tested were ST-11. ST-11 Clonal complex (cc)11 is known to be a hyperinvasive lineage which may replace the Nm A ST-7 lineage since the country was entirely vaccinated with MenAfriVac® at the end of 2011. Further studies are needed to compare the recent Nm W isolates belonging to cc11 and those of 2002-2003 in order to understand the reemergence of this Nm W strain.

We observed a significant difference in the age groups of patients infected with serogroup W or A, with the proportion of serogroup W infections being significantly higher among young children including those under two years of age, than among older subjects. Similarly, the attack rate during the meningitis serogroup W outbreak in Burkina Faso in 2002 was highest in patients <5 years of age and decreased with age [27]. These findings have implications regarding vaccination strategy. Currently, the reactive outbreak response strategy with either the PS A/C vaccine or the PS A/C/W vaccine is usually restricted to those aged 2-29 years because of concerns over the poor responsiveness of infants to polysaccharide vaccines. Use of conjugate vaccines, which are more immunogenic than polysaccharide vaccines in infants, in epidemic responses would allow the whole population to be vaccinated effectively.

## Conclusions

Whereas large outbreaks of Nm A should be eliminated by widespread deployment of the conjugate A vaccine, it is not yet clear how the other serogroups, in particular W, will evolve in response to this new situation. This is an important reason for strengthening surveillance in order to detect and investigate outbreaks, to assess the dynamic of Nm W outbreaks and to adapt the response strategy accordingly. Finally, it is also important to monitor the evolution and distribution of Nm of other serogroups in order to provide information on future vaccine needs. In the medium term, a new, affordable, multivalent (ACWXY) conjugate vaccine may be needed as the currently available quadrivalent meningococcal conjugate ACWY vaccines are too expensive for wide use in Africa.

## Abbreviations

CERMES: Centre de Recherche Médicale et Sanitaire; CSF: Cerebrospinal fluid; DSRE: Direction of Surveillance and Response to Epidemics; HCCA: Health centre catchment areas; MoPH: Ministry of public health; MLST: Multi-locus sequence typing; MVP: Meningitis Vaccine Project; PS: Polysaccharide vaccines; TI: Trans-Isolate medium; WHO: World Health Organization; WHOCC: WHO collaborative centre.

## Competing interests

The authors have read the journal’s policy and have the following conflicts. Funding was received from Sanofi Pasteur; however, there are no patents, products in development or marketed products to declare. This does not alter our adherence to all the BMC Infectious diseases’ policies on sharing data and materials, as detailed online in the guide for authors. Stéphane Hugonnet is a staff member of the World Health Organization. The views expressed in this publication are those of the author alone and they do not necessarily represent the decisions, policy or views of the World Health Organization.

## Authors’ contributions

Analyzed the data: JMC JFJ ZM; Wrote the paper: JMC JFJ BG SH; Epidemiological surveillance and vaccine responses: ZM SH; Laboratory analyses coordinator: BI; Molecular analyses: PN MKT; Map realization: JFJ. All authors read and approved the final manuscript.

## Pre-publication history

The pre-publication history for this paper can be accessed here:

http://www.biomedcentral.com/1471-2334/13/576/prepub
